# Novel platform for engineering stable and effective vaccines against botulinum neurotoxins A, B and E

**DOI:** 10.3389/fimmu.2024.1469919

**Published:** 2024-09-09

**Authors:** Yang Liu, Xiaoyu Liu, Weiwei Chen, Yunzhou Yu, Jianghui Meng, Jiafu Wang

**Affiliations:** ^1^ School of Life Sciences, Henan University, Kaifeng, China; ^2^ Department of Protein Engineering, Beijing Institute of Biotechnology, Beijing, China; ^3^ Pharmaceutical College, Henan University, Kaifeng, China; ^4^ School of Biotechnology, Dublin City University, Dublin, Ireland

**Keywords:** vaccine, botulinum neurotoxin, streptavidin, botulism, therapeutics, protein engineering

## Abstract

Botulinum neurotoxin (BoNT), produced by *Clostridium botulinum*, is the most toxic protein known, capable of causing severe paralysis and posing a significant bioterrorism threat due to its extreme lethality even in minute quantities. Despite this, there are currently no FDA-approved vaccines for widespread public use. To address this urgent need, we have developed an innovative vaccine platform by fusing the neuronal binding domain of BoNT/E (Hc/E) with core-streptavidin (CS), resulting in a stable CS-Hc/E vaccine. Mice vaccinated with CS-Hc/E exhibited superior antibody titers compared to those receiving Hc/E alone. To develop a trivalent vaccine against BoNT/A, BoNT/B, and BoNT/E— key contributors to the vast majority of human botulism—we conjugated CS-Hc/E with a biotinylated atoxic chimeric protein incorporating neutralizing epitopes from BoNT/A and BoNT/B. This chimeric protein includes the binding domain of BoNT/A, along with the protease-inactive light chain and translocation domains of BoNT/B. The interaction between CS and biotin formed a stable tetrameric antigen, EBA. Vaccination with EBA in mice elicited robust antibody responses and provided complete protection against lethal doses of BoNT/A, BoNT/B, and BoNT/E. Our findings highlight EBA’s potential as a stable and effective broad-spectrum vaccine against BoNT. Moreover, our technology offers a versatile platform for developing multivalent, stable vaccines targeting various biological threats by substituting the BoNT domain(s) with neutralizing epitopes from other life-threatening pathogens, thereby enhancing public health preparedness and biodefense strategies.

## Introduction

Botulinum neurotoxins (BoNTs) are the most toxic proteins known to humans and are classified as category A toxins. The estimated median lethal dose of BoNT/A for humans is ~1 ng/kg intravenously or intramuscularly, and ~10 ng/kg when inhaled (1). BoNTs are produced by the bacterium *Clostridium botulinum* and exist in seven immunologically distinct serotypes (/A to/G) in nature. Most BoNT serotypes have multiple subtypes based on variations in their amino acid sequences (2). Each BoNT molecule is ~150 kDa, consisting of a 50 kDa N-terminal protease light chain (LC) and a C-terminal 100 kDa heavy chain (HC) linked by a disulfide bond. The C-terminal nontoxic 50 kDa of HC (Hc) contains two subdomains responsible for binding to neuronal receptors and facilitating receptor-mediated internalization into neurons (3). The N-terminal half of the HC (Hn) forms a channel on the synaptic vesicle/endosomal membrane to transfer the attached LC into the cytosol (4). The intracellular reducing environment cleaves the disulfide bond, releasing the LC, which then acts as the toxic domain. The LC cleaves soluble N-ethylmaleimide-sensitive factor attachment protein receptors (SNAREs), inhibiting synaptic vesicle fusion and transmitter release (2, 5–11). This blockade of acetylcholine release at neuromuscular junctions causes botulism, a severe and potentially fatal illness characterized by muscle paralysis. While botulism is rare, it can occur through foodborne, wound, or infant infections, and inhalational botulism presents a significant bioterrorism threat. Among the BoNT serotypes, A, B, E, and occasionally F are primarily responsible for natural human cases (12, 13).

The dual significance of BoNTs in both medical and bioterrorism contexts has driven extensive research into developing effective countermeasures, such as vaccines and antidotes. For high-risk populations, particularly certain laboratory researchers and military personnel, vaccination has been a key strategy to confer immunity and prevent botulism. Since 1965, the Centers for Disease Control and Prevention (CDC) has provided an investigational formaldehyde-inactivated pentavalent BoNT toxoid to these groups (14). While effective, these vaccines faced limitations, such as production variability, potential residual toxicity, declining potency and logistical challenges related to large-scale manufacturing, leading to their discontinuation for vaccinating workers at risk of occupational exposure since 2011 (15). In contrast, given the therapeutic use of minute amounts of BoNT in treating hyperactive neurological disorders and in cosmetic applications, widespread vaccination of the general population is not recommended unless there is an immediate bioterrorism threat. Currently, no human vaccine for BoNTs has received approval.

Recent advances in recombinant DNA technology have revolutionized BoNT vaccine development by enabling the production of non-toxic derivatives that retain immunogenic properties. Recombinant protease mutant full-length BoNTs and subunit vaccines, which typically target the Hc domain, have been developed as potential vaccines because they stimulate neutralizing antibodies without the risk of toxicity (16–30). Additionally, previous findings also demonstrated that the LCHn domain of BoNTs induces neutralizing antibodies and protects mice from BoNT challenge (31–33).

Given the threat of BoNTs as bioterrorism agents, there is an urgent need for diverse vaccine platforms and delivery systems that are safer, more effective, and more stable. Towards this, we have engineered a novel, stable tetrameric antigen, EBA, which incorporates functional neutralising epitopes from BoNT/A, BoNT/B, and BoNT/E through core-streptavidin (CS) and biotin interactions. Vaccination with EBA in mice generated robust neutralizing antibody responses and provided complete protection against lethal doses of these toxins. EBA shows promise as a stable, effective broad-spectrum BoNT vaccine and offers adaptability for combating other biological threats.

## Material and methods

### Materials

pMAL-C4X, pET-29a, and pGEX-4T-2 vectors were purchased from BGI Genomics Co., Ltd. (Beijing, China). BL21.DE3 chemically competent cells (Catalog no. TSC-E01) were purchased from Tsingke Biotech Co., Ltd. (Beijing, China). Ampicillin (Catalog no. A8180), kanamycin (Catalog no. K8020), horseradish peroxidase (HRP) conjugated rabbit anti-horse IgG (Catalog no. SE242), and 3,3′,5,5′-tetramethylbenzidine (TMB) single-component substrate solution (Catalog no. PR1200) were purchased from Beijing Solarbio Science & Technology Co., Ltd. Talon super-flow metal affinity resin (Catalog no. 635670) was purchased from Takara Biomedical Technology Co., Ltd. (Beijing, China). Amylose resin and restriction enzymes were purchased from New England Biolabs Co., Ltd. (Beijing, China). HRP-conjugated anti-rabbit or anti-mouse secondary antibodies and mouse anti-MBP-tag mAb antibodies (Catalog no. AE016, 1:5000 dilution) were purchased from ABclonal Technology Co., Ltd. (Wuhan, China). Tryptone (Catalog no. LP0042B), yeast extract (Catalog no. LP0042B), D-biotin (Catalog no. B20656), ECL Western blotting substrate (Catalog no. 32209), and alum adjuvant (Catalog no. 77161) were purchased from Thermo Fisher (Shanghai, China). PD-10 desalting columns (Catalog no. 17085101) were obtained from Cytiva Lifesciences (Shanghai, China). Thrombin (Catalog no. T69671), benzonase (Catalog no. 70746) and a protease inhibitor cocktail III (Catalog no. 539134) were purchased from Millipore (Shanghai, China). Routinely used chemicals were sourced from Sigma-Aldrich (Shanghai, China). Horse anti-Hc/A, Hc/E and LC.Hn/B were reported previously (32, 34). The BoNT/A, BoNT/B and BoNT/E were sourced from National Institutes of Food and Drug Control, Beijing, China.

### Animals

Female SPF Balb/C mice (6-8 weeks old) and female Kunming (KM) mice (15–18 g) were provided by the Beijing Vital River Laboratory Animal Technology Co., Ltd (Beijing, China) and SiPeiFu (SPF) Biotechnology Co., Ltd. (Beijing, China). The animals were randomly assigned to different groups and housed in the pathogen-free bioresource unit under a 12h light/dark cycle at 22°C and ~50% humidity, with ad libitum access to food and water. For euthanasia, mice were exposed to carbon dioxide inhalation, introduced at a rate of at least 30% chamber volume per minute. This was followed by cervical dislocation. For anesthesia, mice were placed in the induction chamber before administering isoflurane at a level of 3-4% for induction and 1-2% for maintenance. The oxygen flow rate was adjusted to 1 L/min. Group sizes used in these studies are comparable to similar studies previously reported (32).

### Preparation of recombinant CS-Hc/E fusion protein

To prepare CS-Hc/E, the original pMAL-C4X vector (BGI Genomics Co., Ltd.) was modified by replacing the factor X site with a thrombin recognition site followed by a Bam HI restriction site, resulting in the new vector pMAL-C4T. The codon-optimized synthetic gene (BGI Genomics Co., Ltd.) encoding CS-Hc/E with a thrombin removable His_6_ tag was cloned into the pMAL-C4T plasmid between Bam HI and Hind III sites. The constructed vector was transformed into BL21.DE3 competent cells for expression following established protocol (35). Briefly, bacteria cultured overnight in LB medium with 100 μg/ml ampicillin were inoculated (1:1000 v/v) into ZYP-5052 auto-induction medium (36) supplemented with 100 μg/ml ampicillin. The culture was incubated at 37°C with shaking (220 rpm) for 5 hours, followed by 22°C for 20 hours. Cells were harvested by centrifugation at 8000 g for 30 minutes. The cell pellet from 1 L of culture was resuspended in 40 mL of lysis buffer (20 mM HEPES, 145 mM NaCl, pH 8.0) containing a protease inhibitor cocktail III (1:200, v/v). Lysozyme (final concentration 2 mg/ml), PMSF (1 mM), and 750 units of Benzonase nuclease were added, and the mixture was incubated at 4°C for 1 hour before storage at -80°C. After one freeze-thaw cycle, the lysate was centrifuged at 18000 rpm at 4°C for 1 hour to remove cell debris. CS-Hc/E protein in the supernatant was purified by immobilized metal affinity chromatography (IMAC) using Talon super-flow resin following the manufacturer’s protocols. The eluted sample was buffer exchanged using a PD-10 column to 20 mM HEPES, 145 mM NaCl, pH 7.4.

### Engineering biotinylated protease-inactive chimeric protein of BoNT/B and BoNT/A) (Bio-BoTIM/BA)

The codon-optimized synthetic gene encoding LC(E_231_QH_234_Y)Hn/B-Hc/A with an N-terminal Avi-tag and an C-terminal His_6_ tag (BoTIM/BA) was subcloned into the pET29a vector between the Nde I and Xho I sites. Simultaneously, the synthetic biotin ligase (BirA) gene was cloned into the pGEX-4T-2 vector between the Bam HI and Not I restriction sites. For *in vivo* biotinylation of the BoTIM/BA protein, the BoTIM/BA and BirA expression vectors were co-transformed into BL21.DE3 cells. The overnight culture was inoculated into ZYP-5052 autoinduction medium supplemented with 100 μg/ml kanamycin and 100 μg/ml ampicillin. After 5 hours of incubation at 37°C with shaking, 1 ml of 50 mM D-biotin was added to 500 ml of culture before reducing the temperature to 22°C for an additional 20 hours. The His_6_-tagged Bio-BoTIM/BA was purified by IMAC using Talon resin.

### Cross-linking of CS-Hc/E and Bio-BoTIM/BA protein to produce EBA antigen

The IMAC-purified CS-Hc/E protein was loaded onto an amylose resin column and washed three times with five resin volumes of HEPES buffer (20 mM HEPES, 145 mM NaCl, pH 7.4). Purified Bio-BoTIM/BA protein was then added to the column, and the flow-through was reloaded onto the column twice to facilitate binding of Bio-BoTIM/BA to CS-Hc/E. Contaminants and excess unbound BoTIM/BA protein were removed by extensive washing with HEPES buffer. The cross-linked protein complex was eluted with HEPES buffer containing 10 mM maltose. To remove the MBP and His_6_ tags from CS-Hc/E, the eluted protein was incubated with thrombin (1 unit/mg of protein) for 3 hours at 22°C, followed by inhibition with 1 mM PMSF. The thrombin-treated sample was then purified further by IMAC and buffer exchanged to HEPES buffer to yield the final tetrameric cross-linked EBA antigen.

### SDS-PAGE and western blotting

Samples from each purification step, with and without boiling, were analyzed by SDS-PAGE on 10% Bis-Tris gels and stained with Coomassie Blue. For Western blotting, proteins separated by SDS-PAGE were transferred onto PVDF membranes, blocked for 1 hour at 22°C in TBST buffer containing 5% milk, and then incubated overnight at 4°C with horse anti-Hc/A (1:5000), Hc/E (1:5000), or BoNT/B (1:500) serum. After thorough washing, the membranes were probed with HRP-conjugated secondary antibodies (1:10000). Following three additional washes with TBST buffer, the membranes were subjected to chemiluminescent detection or developed with TMB blotting solution, and protein bands were visualized using the G:BOX Chemi-16 gel documentation system (Syngene G: BOX Chemi XX9, Syngene, UK).

### Immunization with CS-Hc/E, Hc/E, EBA, and BoNT challenge

Female SPF Balb/C mice (6-8 weeks old, Beijing Biotechnology) were randomly assigned to different immunization groups (5-8 mice per group). Purified recombinant proteins CS-Hc/E, Hc/E, or EBA were diluted in PBS and formulated with an equal volume of alum adjuvant. Intramuscular injections of various doses of the recombinant proteins were administered to mice at two-week intervals (50 μl per mouse). For CS-Hc/E and Hc/E samples, 8 mice were used per sample per dose; for EBA, 5-8 mice per group were used. Mice injected with PBS and alum adjuvant served as the control group. Blood samples were collected from tail before each immunization and 14 days after the final immunization to obtain sera, which were used to determine the titers of neutralizing antibodies against the respective toxin serotypes and total antibodies against the individual domains of BoNTs.

For the BoNT challenge experiments, three weeks after two or three vaccinations with 1 µg or 10 µg of EBA protein, or PBS control, the mice were anesthetized and intraperitoneally injected with 200 µl of saline solution containing 10^2^ or 10^3^ LD_50_ doses of BoNT/A, BoNT/B, or BoNT/E (for BoNT/A and BoNT/B group, 8 mice per group; for BoNT/E group, 8 mice per group except 5 mice used for vaccination twice group). The survival of mice in all groups was monitored for one week, and survival rates were recorded.

### Determination of serum antibody titers

Enzyme-linked immunosorbent assay (ELISA) was employed to quantify anti-Hc/A, Hc/E, and LCHn/B antibody levels in the serum of mice immunized with the EBA antigen. Briefly, 96-well ELISA plates (Corning Incorporated, NY, USA) were coated with 100 μl of Hc/A, Hc/E, or LCHn/B (2 µg/ml) and incubated overnight at 4°C. The plates were then incubated with 200 μl of PBS containing 2% skim milk blocking buffer at 37°C for 2 hours before washing with PBS containing 0.1% Tween 20 (PBST). Serum samples were initially diluted 1:100 in blocking buffer and subjected to a two or four-fold serial dilution. Diluted serum samples (100 µl/well) were added to the washed plate and incubated at 37°C for 1.5 hours. After extensive washing with PBST, plates were incubated with 100 μl of diluted HRP-conjugated goat anti-mouse IgG (1:5000) at 37°C for 30 minutes. Following washing with PBST, the plates were developed using 50 μl of citrate buffer (pH 5.0) containing 0.04% (w/v) o-phenylenediamine and 0.02% (v/v) hydrogen peroxide. The reaction was stopped with 50 μl of 2 M H_2_SO_4_, and absorbance was read at 492 nm. For the detection of anti-Hc/E antibody titers in the serum of mice immunized with CS-Hc/E or Hc/E, 100 μl of TMB single-component substrate solution was added and incubated for 10 minutes at room temperature, followed by the addition of 100 μl of 1 M H_2_SO_4_ to stop the reaction. Absorbance was measured at 450 nm using a microplate reader. Antibody titers were determined as the reciprocal of the highest serum dilution yielding an absorbance reading exceeding 0.5 units and the ratio of the specific absorbance of the antigen vaccination group to the PBS control group exceeding 2.

### Determination of BoNT serotype specific neutralizing antibody titers

The neutralizing potency of mouse sera, obtained from mice vaccinated three times with 1 µg or 10 µg of EBA, was evaluated using our established method (37). Briefly, 500 µl of serially diluted serum samples were mixed with 1 ml of a standard concentration of BoNT/A, BoNT/B, or BoNT/E, each containing 100 LD_50_ units of toxin. Subsequently, 1 ml of PBS was added to achieve a total volume of 2.5 ml. The mixtures were incubated for 1 hour at room temperature to allow antibody binding to the neurotoxin. These samples were then intraperitoneally injected into anesthetized female KM mice weighing 15–18 g (500 µl/mouse, 4 mice per group). Survival of the mice was monitored for 1 week starting from 4 hours post-injection. Serum neutralizing potency was expressed in international units per ml (IU/ml), in accordance with the World Health Organization (WHO) standards for BoNT/A,/B, and/E antitoxins. An IU for BoNT/A and BoNT/B is defined as the neutralizing antibody amount that can neutralize 10^4^ mouse LD_50_ of BoNTs, whereas for serotype E, it neutralizes 10^3^ mouse LD_50_ of BoNT/E.

### Statistical analysis

Statistical analysis and data graphing were performed using Prism 9 software (GraphPad Software, San Diego, California, USA). All data are presented as means ± SEMs, with sample sizes (n) specified in the figure or legends. No experimental units or data points were excluded from the analysis. Statistical differences in the antibody titers between groups were determined using one-way ANOVA followed by Tukey’s *post hoc* test or an unpaired two tailed Student’s t test. A probability value of less than 0.05 was considered statistically significant. NS denotes non-significant results with p > 0.05, while * indicates p < 0.05, ** indicates p < 0.01, and *** indicates p < 0.001.

## Results

### Designing EBA trivalent vaccine for broad-spectrum protection against BoNT/A,/B and/E

**Figure 1:**
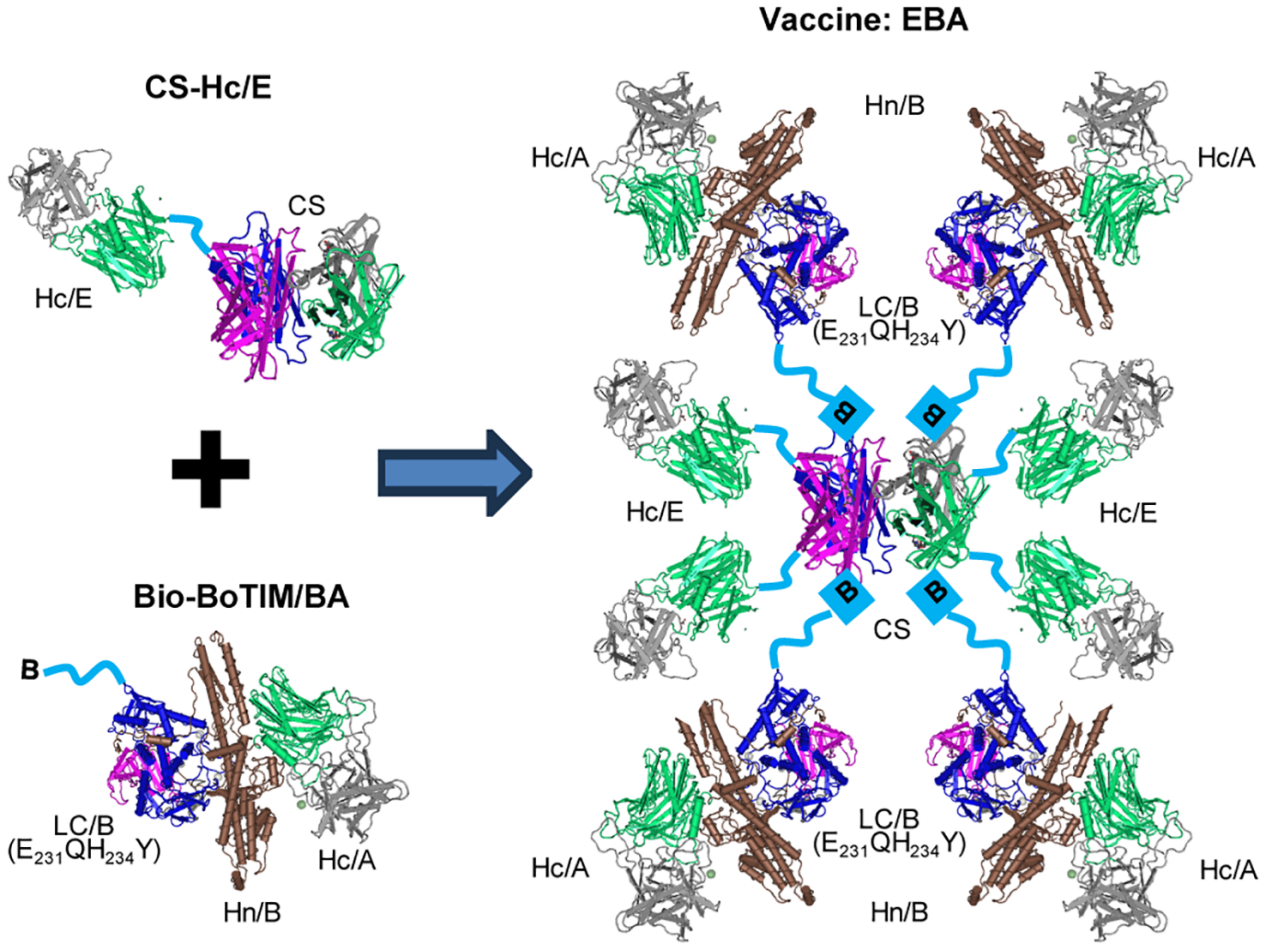
Design strategy of EBA vaccine. The schematic illustrates the method for engineering the large, stable tetrameric molecule, EBA. This involves conjugating a recombinant CS-Hc/E fusion protein to site-directed biotinylated BoTIM/BA (Bio-BoTIM/BA) to create EBA. The symbol B linked to LC/B(E231QH234Y) represents biotin. The crystal structure of CS was adapted from (53), while the crystal structure of BoNT/A (54) was used to depict the individual domains of LC, Hn, and Hc, serving as a general illustration rather than representing specific BoNT serotypes.

In developing a stable and effective vaccine against BoNT/A, BoNT/B, and BoNT/E, we first designed a novel CS-Hc/E fusion protein, which forms a tetrameric core protein via the properties of CS (**Figure 1**). Second, we exploited key protective antigens from the LCHn domain of BoNT/B and the Hc domain of BoNT/A to create an avi-tagged, protease-inactive, atoxic chimeric protein BoTIM/BA. In BoTIM/BA, two key residues in the highly conserved zinc-binding motif HEXXH were mutated (E_231_QH_234_Y) to remove its SNARE-cleaving activity. Third, the CS moiety in the core platform can bind four Bio-BoTIM/BA antigens to form a large, stable tetrameric molecule (termed EBA) (**Figure 1**).

### Protein engineering and characterization of the CS-Hc/E and EBA

**Figure 2:**
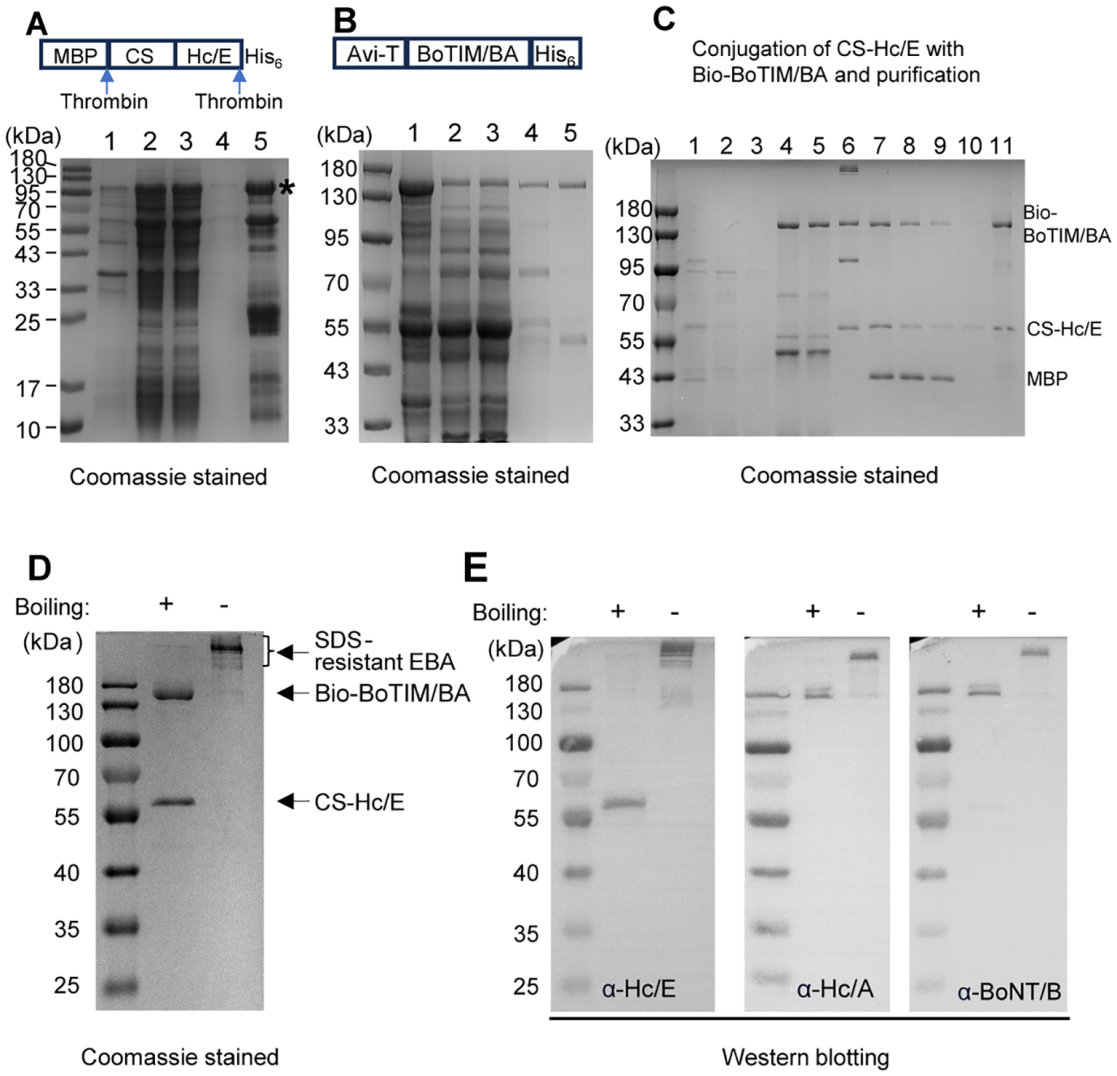
Expression and purification of CS-Hc/E and Bio-BoTIM/BA and their conjugation to yield EBA. **(A)** SDS-PAGE analysis of aliquots (boiled) from IMAC purification of CS-Hc/E. Lanes: 1, insoluble fraction after lysis; 2, soluble fraction; 3, flow through from IMAC column; 4, wash fraction; 5, eluted fraction. *, labelling the intact MBP-CS-Hc/E-His_6_ protein. **(B)** IMAC purification of Bio-BoTIM/BA. Lanes: 1, insoluble fraction after lysis; 2, soluble fraction; 3, flow through from IMAC column; 4, wash fraction; 5, eluted fraction. **(C)** Conjugation of CS-Hc/E with Bio-BoTIMA/BA followed by purification and removal of MBP tag (details see Methods). Lanes: 1, IMAC purified CS-Hc/E; 2, flow-through from amylose column; 3, wash fraction from amylose column**;** 4, flow-through from amylose column after loading excess Bio-BoTIM/BA; 5, wash fraction; 6, elute from amylose column; 7, thrombin treated conjugate; 8, flow-through of thrombin treated conjugate from IMAC; 9, 10, wash fractions; 11, final eluted EBA sample. Note, all samples were boiled. **(D)** Final purified EBA protein was subjected to SDS-PAGE followed by Coomassie staining. Note that, pre-boiling of EBA samples is required to separate its components even in the SDS-PAGE. **(E)** Western blots verified the components of EBA with specific antibodies against BoNT/B, Hc/E, or Hc/A domain.

To engineer the EBA protein, we inserted a synthetic gene encoding CS-Hc/E with a C-terminal thrombin-removable His_6_ tag into our modified pMAL-C4T vector, which contains a thrombin-removable N-terminal MBP tag. This resulted in a construct encoding the MBP-CS-Hc/E-His_6_ fusion protein (**Figure 2A**). This recombinant protein was expressed in BL21.DE3 cells using auto-induction medium and partially purified using IMAC on Talon resin (**Figure 2A**). The purified protein displayed the expected monomer band with a molecular weight of ~110 kDa after boiling (**Figure 2A**). Nearly all contaminants were removed during the later conjugation process.

To engineer site-specific biotinylated BoTIM/BA, a synthetic gene encoding the N-terminal avi-tagged LC(E_231_QH_234_Y)Hn/B-Hc/A-His_6_ fusion protein was inserted into the pET29a vector. Similarly, a synthetic gene encoding biotin-protein ligase (BirA) was cloned into the pGEX-4T2 vector. For expression and biotinylation of BoTIM/BA, sequence-verified plasmids expressing His_6_-tagged BoTIM/BA and GST-fused BirA were co-transformed into BL21.DE3 cells. Biotin-labelled BoTIM/BA was expressed inside *E. coli* using auto-induction medium supplemented with biotin. Bio-BoTIM/BA protein was purified with an expected molecular weight of ~150 kDa by IMAC (**Figure 2B**).

To couple the Bio-BoTIM/BA to CS-Hc/E, IMAC-purified MBP-CS-Hc/E-His_6_ was loaded onto an amylose resin column. After extensive washing to remove contaminants, excess Bio-BoTIM/BA was applied to the column, and unbound Bio-BoTIM/BA and contaminants were washed away. The tagged BoTIM/BA-CS-Hc/E complexes were eluted with maltose. Subsequently, the MBP and His_6_ tags were removed from the core protein by incubation with thrombin (**Figure 2C**). The final tetrameric molecule (EBA) was purified by IMAC, with the removal of the MBP tag (**Figure 2C**). SDS-PAGE analysis showed that the majority of the EBA conjugate remained stable in urea and SDS-resistant complex forms, even when subjected to heating at temperatures up to 60°C (**Figure 2D**; **Supplementary Figure S1**). It was only after boiling for 10 minutes that its components, Bio-BoTIM/BA and CS-Hc/E, separated during SDS-PAGE (**Figure 2D**). Western blot analysis of EBA confirmed the homogeneity of the recombinant proteins (**Figure 2E**). Antibodies against Hc/A or BoNT/B recognized the Bio-BoTIM/BA protein, while Hc/E specifically visualized the fragment corresponding to CS-Hc/E, confirming the components of the final purified EBA (**Figure 2E**).

To verify the significant reduction in neurotoxicity upon mutating key residues in the LC of BoTIM/BA, we intraperitoneally injected 50 µg of EBA into mice. No signs of botulism were observed during the 7-day observation period, and there was no impact on body weight (data not shown).

### CS-Hc/E vaccinated mice exhibit enhanced immune response compared to Hc/E alone

**Figure 3:**
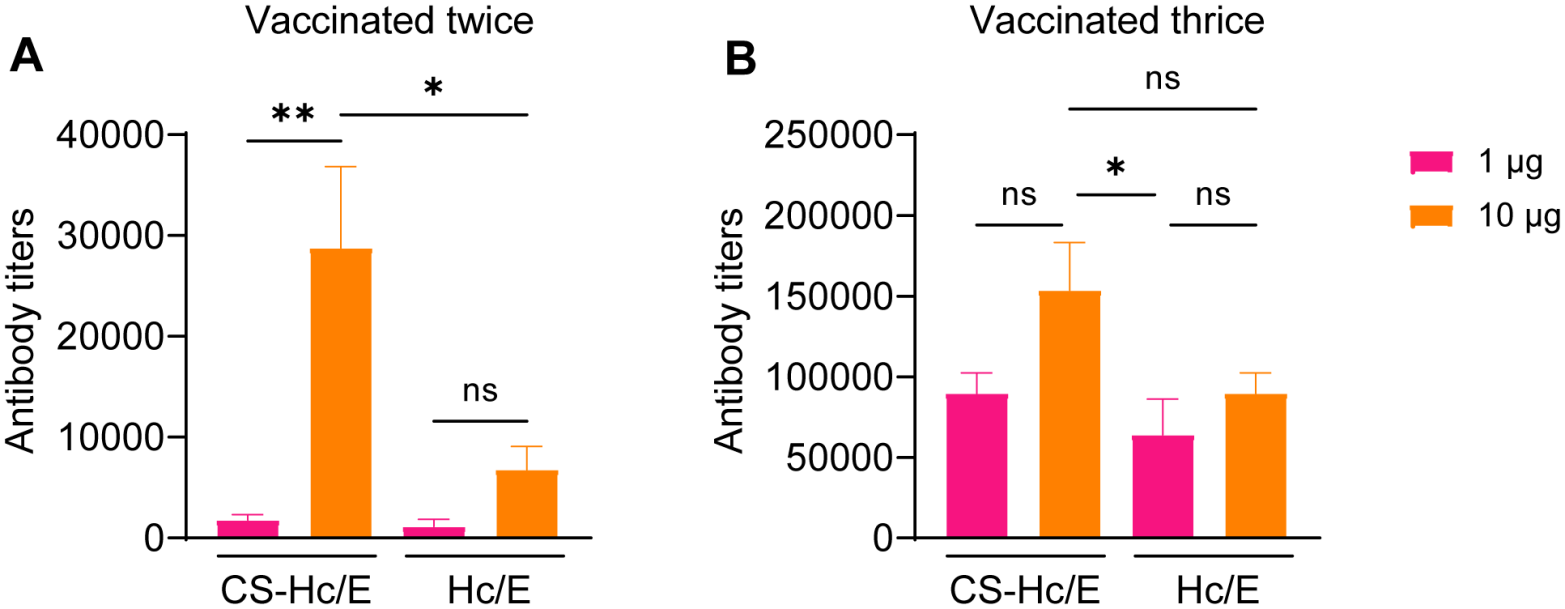
Serum antibody titers in mice immunized with CS-Hc/E or Hc/E. Antibody titers against Hc/E in the serum samples from mice vaccinated twice **(A)** or three times **(B)** with 1 µg or 10 µg recombinant Hc/E or CS-Hc/E antigens were determined by ELISA. Data are mean ± SEM, n=8 mice. Two mouse serum samples were combined for ELISA. *, p<0.05; **, p<0.01; ns, non-significant; one-way ANOVA.

We initially assessed whether the fusion of CS to Hc/E affected its efficacy as an antigen. Mice were vaccinated twice or three times with either 1 µg or 10 µg of CS-Hc/E. Equivalent doses of Hc/E and vehicle control PBS were also administered to separate groups of mice for comparison. Blood samples were collected before each injection to measure antibody titers by ELISA. After two immunizations with 1 µg of CS-Hc/E or Hc/E antigens, both induced similar specific antibody responses (**Figure 3A**). Antibody titers were significantly increased to comparable levels following an additional immunization (**Figure 3B** vs. **Figure 3A**). Notably, two immunizations with 10 µg of CS-Hc/E resulted in much higher antibody titers compared to Hc/E (p<0.05) (**Figure 3A**). Additionally, a trend towards higher average antibody titers was observed after three immunizations with 10 µg of CS-Hc/E compared to Hc/E (**Figure 3B**). These results demonstrate that the fusion of CS to Hc/E enhances its antigenic efficacy. Interestingly, increasing the dose of CS-Hc/E or Hc/E from 1 µg to 10 µg did not significantly boost the specific antibody titer if mice were vaccinated three times (**Figure 3B**).

### EBA vaccination in mice elicits robust antibody responses and provides complete protection in mice against lethal doses of BoNT/A, BoNT/B, and BoNT/E

**Figure 4:**
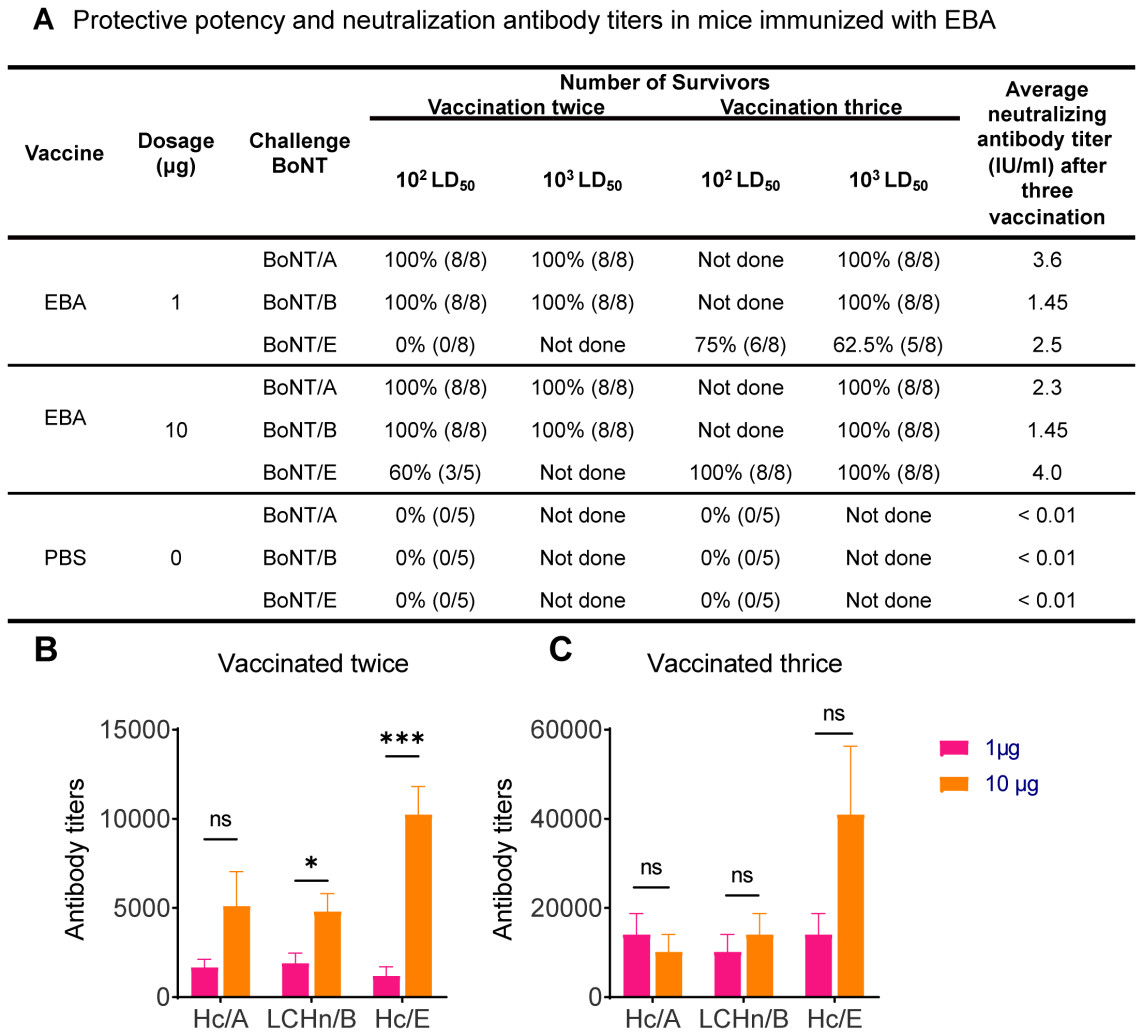
Determination of serum antibody titers in mice immunized with EBA and protective potency of EBA antigen. **(A)** Protective potency and neutralization antibody titers in mice immunized twice or thrice with 1 µg or 10 µg EBA, PBS and alum adjuvant formulation was injected as negative control. **(B, C)** Graphs showing the specific antibody titers against BoNT/A,/B and/E specific domains in the serum samples from mice vaccinated twice **(B)** or thrice **(C)** with 1 µg or 10 µg EBA antigen. Data are mean ± SEM, n=5-8 mice. *, p<0.05; ***, p<0.001; ns, non-significant; unpaired two tailed Student’s t-test.

Next, we evaluated the immunoprotective efficacy of the EBA protein as an antigen against toxins. Mice immunized with EBA were challenged with 10^2^ or 10^3^ LD_50_ doses of BoNT/A, BoNT/B, or BoNT/E. Our results demonstrated that two immunizations with 1 µg of EBA provided complete protection for mice against challenges with 10^3^ LD_50_ BoNT/A or BoNT/B (**Figure 4A**). Given that the ratios of Hc/A and LCHn/B in EBA are less than 1/4 and 1/2, respectively, our findings confirm that Hc/A and LCHn/B components in EBA have strong immunogenic potency, generating neutralizing antibodies against BoNT/A and BoNT/B. However, only a majority of mice (62.5%) vaccinated three times with 1 µg of EBA survived the challenge with 10^3^ LD_50_ BoNT/E (**Figure 4A**). Nevertheless, 60% of mice immunized twice with 10 µg of EBA survived the challenge with 10^2^ LD_50_ BoNT/E; one additional immunization provided complete protection for mice from the higher dose (10^3^ LD_50_) BoNT/E challenge (**Figure 4A**).

We further evaluated the neutralizing antibody titers. Serially diluted serum from mice immunized three times with 1 µg or 10 µg of EBA was mixed with a standard concentration (100 LD_50_/ml) of BoNT/A, BoNT/B, or BoNT/E to allow the antibody to bind the specific epitopes of the neurotoxin before intraperitoneal injection into mice. The neutralizing antibody response produced by the 1 µg EBA antigen for BoNT/A, BoNT/B, and BoNT/E was 3.6, 1.45, and 2.5 IU/ml, respectively (**Figure 4A**). The neutralizing antibody titers against BoNT/E increased when the vaccination dose was increased from 1 µg to 10 µg (**Figure 4A**). There was no change in neutralizing antibody titers against BoNT/B between the groups vaccinated with 1 µg and 10 µg (**Figure 4A**). Nevertheless, our results confirmed that all vaccination doses displayed a high level of protective potency. This establishes that EBA is an effective antigen for generating antidotes against BoNT/A, BoNT/B, and BoNT/E.

Next, we measured the specific antibody titers in the serum of mice following EBA vaccinations. ELISA plates were coated with recombinant Hc/A, LCHn/B, or Hc/E proteins to quantify the BoNT serotype-specific antibody titers. Two immunizations with 1 µg of EBA antigen induced a strong specific antibody response against the Hc/A, LCHn/B, and Hc/E domains (**Figure 4B**). Increasing the vaccination dose from 1 µg to 10 µg enhanced the specific antibody titers against Hc/A and LCHn/B, with a pronounced effect (over 8.5-fold) on antibodies against Hc/E (**Figure 4B**). This is consistent with our earlier observation that two immunizations of 10 µg of CS-HcE generated more than 16-fold higher antibody levels compared to 1 µg of CS-HcE (cf. **Figure 3A**). We also measured the antibody titers after three immunizations with 1 µg of EBA. The average antibody titers against Hc/A, LCHn/B, and Hc/E increased by 8.4, 5.3, and 11.7-fold, respectively, compared to two vaccinations with the same dose of EBA (**Figure 4C** vs **Figure 4B**). In contrast, one additional immunization with 10 µg of EBA enhanced the specific antibody titers against Hc/A, LCHn/B, and Hc/E by 2, 2.9, and 4-fold, respectively (**Figure 4C** vs **Figure 4B**). Notably, we found no increase in antibody titers against Hc/A and LCHn/B between groups vaccinated three times with 1 µg and 10 µg of EBA (p>0.05) (**Figure 4C**), suggesting that increasing the antigen dose does not elevate antibody titers when three vaccinations are used to protect against BoNT/A and BoNT/B challenges. However, average antibody titers against Hc/E increased further with higher antigen doses (**Figure 4C**), explaining why three vaccinations with 10 µg of EBA can protect all mice challenged with 10^3^ LD_50_ BoNT/E, compared to partial protection with 1 µg of EBA (cf. **Figure 4A**).

## Discussion

The present study demonstrates the development and evaluation of an innovative multivalent vaccine platform targeting BoNTs. By leveraging the strong interaction between CS and biotin, we engineered a stable tetrameric antigen (EBA) that incorporates key neutralizing epitopes from BoNT/A, BoNT/B, and BoNT/E. Our findings highlight the potential of EBA as a broad-spectrum vaccine candidate against BoNTs, which pose significant threats due to their extreme toxicity and potential use in bioterrorism.

Our initial focus was on the efficacy of the CS-Hc/E fusion protein. By conjugating the Hc/E to CS, we hypothesized that multivalent presentation of antigens would enhance the immune response. Our results confirmed that mice vaccinated with CS-Hc/E exhibited significantly higher antibody titers compared to those receiving Hc/E alone. This enhancement in immunogenicity may be attributed to the improved stability and prolonged antigen presentation provided by the CS fusion strategy, consistent with previous findings that multivalent antigen presentation can enhance B cell responses to protein subunit vaccines (38, 39).

To develop a comprehensive vaccine capable of protecting against multiple BoNT serotypes, we engineered an SDS-resistant trivalent antigen, EBA, by coupling the CS-Hc/E to BoTIM/BA. Our previous research demonstrated that the protease-active chimera BoNT/BA exhibits extraordinarily high specific neurotoxicity in mice (6x10^8^ LD_50_ units/mg) (40), confirming that chimera BA possesses all essential features of an active neurotoxin. These features include binding to neuronal receptors through Hc/A, translocation of LC/B protease by Hn/B, cleavage of intracellular neuronal target SNARE proteins, and inhibition of acetylcholine exocytosis, leading to lethality. Our toxicity studies confirmed that EBA, despite containing multiple components derived from highly toxic BoNTs, exhibited no adverse effects in mice after mutating the key residues responsible for its zinc metalloprotease activity, highlighting its safety for potential use in humans.

Vaccination with EBA induced robust antibody responses against all three targeted BoNT serotypes, demonstrating its ability to stimulate a potent humoral response. Importantly, the vaccinated mice were completely protected against lethal doses of BoNT/A, BoNT/B, and BoNT/E. This suggests that the EBA tetrameric structure effectively presents epitopes from BoNT/A, BoNT/B, and BoNT/E, which is critical for eliciting a broad and effective immune response. Like other reported subunits or genetically inactivated holotoxins, the components of the EBA vaccine can be easily produced in *E. coli* and scaled up efficiently, potentially offering a cost-effective and efficacious advantage to DNA and mRNA vaccines for multivalent protection against BoNTs (41–48), which face challenges due to the large coding size of these toxins. Consistent with earlier findings that LC.Hn/A vaccine provides nearly equivalent protection against BoNT/A challenge as inactivated holotoxin, LCHn/B in BoTIM/BA offers excellent protection against BoNT/B challenges. Moreover, our approach of combining epitopes from different serotypes, as seen with naturally occurring chimeric neurotoxins such as BoNT/CD, BoNT/DC, and BoNT/FA (or BoNT/HA, originally reported as BoNT/H) (49–52), addresses antigenic variability among BoNT serotypes, thereby ensuring comprehensive protection which may provide potent protection compared to the mixed Hc or full-length of BoTIM vaccines. Interestingly, the protection against BoNT/E was relatively lower compared to BoNT/A and BoNT/B, despite EBA vaccination inducing higher specific antibody titers against Hc/E. This observation aligns with previous findings that the Hc/E domain provides less immunogenic protection compared to counterparts from other serotypes (16). Future efforts could focus on replacing the Hc/E epitope with a protease-inactive BoNT/E (BoTIM/E) to enhance the efficacy of this trivalent vaccine, as studies have shown that BoTIM/E offers higher immunogenic protection than Hc/E domain alone (16). Alternatively, given that CS can bind four biotinylated molecules, our method could be adapted to include additional biotinylated inactive BoTIMs, such as BoTIM/E, BoTIM/C, BoTIM/D, or BoTIM/F, or their chimeric proteins, as demonstrated with BoTIM/BA.

One of the most promising aspects of our vaccine platform is its versatility. The CS and biotin interaction system used to construct EBA can be adapted to incorporate neutralizing epitopes from various pathogens, not just BoNTs. This adaptability makes our platform a valuable tool for developing multivalent vaccines against a range of biological threats, enhancing public health preparedness and biodefense strategies. Additionally, the platform’s ability to generate strong and broad immune responses also highlights its potential for rapid adaptation and deployment in response to emerging infectious diseases and bioterrorism threats.

In conclusion, our study introduces a novel and effective multivalent vaccine platform capable of providing broad-spectrum protection against BoNT/A, BoNT/B, and BoNT/E, which are responsible for most human botulism cases. The EBA protein demonstrated enhanced immunogenicity, robust antibody responses, and complete protection in mice. The versatility of our platform offers significant potential for developing vaccines against various pathogens, contributing to global efforts in enhancing biodefense and public health preparedness.

## Data Availability

The original contributions presented in the study are included in the article/**Supplementary Material**. Further inquiries can be directed to the corresponding author.
