# Supplementary material for: Novel platform for engineering stable and effective vaccines against botulinum neurotoxins A, B and E
Source: Front Immunol. 2024 Sep 9;15:1469919. doi: 10.3389/fimmu.2024.1469919 (PMC11416995; doi:10.3389/fimmu.2024.1469919)
Supplement: Supplementary file 1 [file DataSheet1.pdf]

Supplementary Figure S1

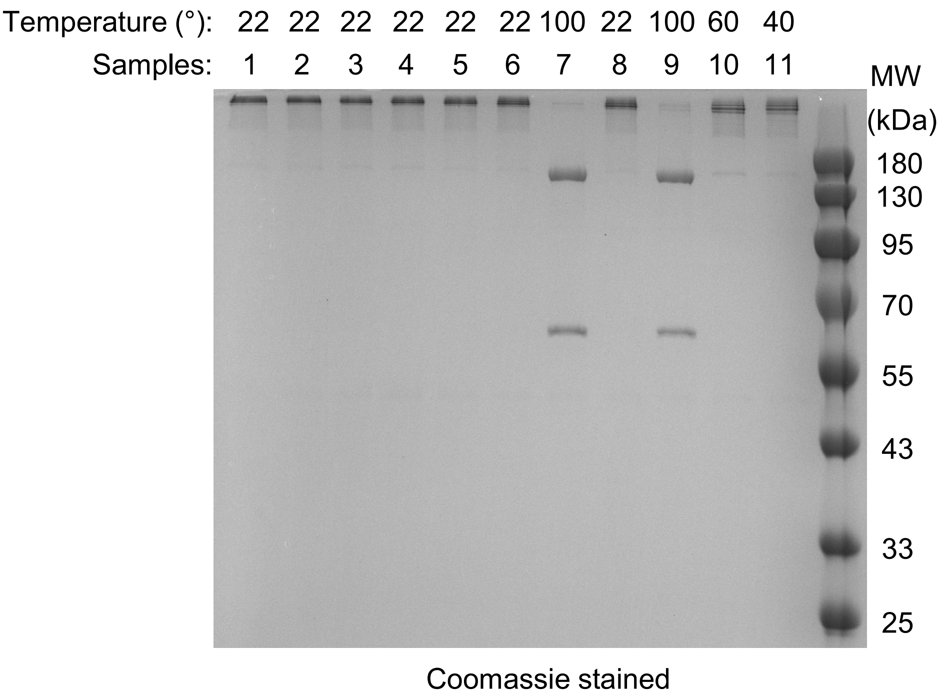

Supplementary Figure S1: SDS-PAGE analysis of EBA vaccine under stressed conditions. Purified EBA proteins were subjected to various stress conditions, including treatment with different concentrations of SDS and urea, or heating up to 100°C for 10 minutes, followed by SDS-PAGE and Coomassie staining. Samples: 1, 3% SDS treatment; 2, 1% SDS treatment; 3, 4, 5: treatment with 6M, 4M, and 2M urea, respectively (samples 1–5 were initially stored at -80°C); 6, sample stored at 4°C for 6 months; 7, sample stored at 4°C for 6 months and then boiled for 10 minutes; 8, sample stored at -80°C; 9, sample stored at -80°C and then boiled for 10 minutes; 10, sample stored at -80°C and heated to 60°C for 10 minutes; 11, sample stored at -80°C and heated to 40°C for 10 minutes.
